# The Role of Netrin-1 in Improving Functional Recovery through Autophagy Stimulation Following Spinal Cord Injury in Rats

**DOI:** 10.3389/fncel.2017.00350

**Published:** 2017-11-03

**Authors:** Liangjie Bai, Xifan Mei, Yanfeng Wang, Yajiang Yuan, Yunlong Bi, Gang Li, Hongyu Wang, Peng Yan, Gang Lv

**Affiliations:** ^1^Department of Orthopedics, The First Affiliated Hospital of China Medical University, Shenyang, China; ^2^Department of Orthopedics, The First Affiliated Hospital of Jinzhou Medical University, Jinzhou, China

**Keywords:** spinal cord injury, Netrin-1, autophagic flux, apoptosis, AMPK, mTOR, transcription factor EB

## Abstract

Our previous findings indicated that treatment with Netrin-1 could improve functional recovery through the stimulation of autophagy, by activating the AMP-activated protein kinase/mammalian target of rapamycin (AMPK/mTOR) signaling pathway in rats following spinal cord injury (SCI). However, the underlying mechanisms were not elucidated. The purpose of this study was to investigate the underlying mechanisms by which Netrin-1 promotes autophagy and improves functional recovery after SCI. Following controlled SCI in Sprague-Dawley rats, we observed that the autophagic flux in neurons was impaired, as reflected by the accumulation of light chain 3-II (LC3-II)-positive and LC3-positive autophagosomes (APs), accompanied by the accumulation of the autophagic substrate, Sequestosome 1 (SQSTM1; also known as p62). Our results showed that treatment with Netrin-1 increases the levels of the lysosomal protease cathepsin D (CTSD) and lysosomal-associated membrane protein 1 (LAMP1), through the regulation of the nuclear localization of Transcription factor EB (TFEB) via the AMPK/mTOR signaling pathway. In addition, this enhancement of lysosomal biogenesis correlated strongly with the restoration of autophagic flux, inhibition of neural apoptosis and improved functional recovery. Suppression of lysosomal biogenesis via the inhibition of the nuclear translocation of TFEB by Compound C abolished this restoration of autophagic flux and the functional recovery effects of Netrin-1 following SCI. Taken together, these results indicate that Netrin-1 enhances lysosomal biogenesis by regulating the nuclear translocation of TFEB via the AMPK/mTOR signaling pathway. Furthermore, the enhancement of lysosomal biogenesis by Netrin-1 following SCI promotes autophagic flux and improves functional recovery in rats. Thus, the regulation of lysosomal biogenesis by modulating the nuclear localization of TFEB might be a novel approach for the treatment of SCI.

## Introduction

Spinal cord injury (SCI) remains a serious health problem worldwide that affects about 273,000 people in the United States alone, and almost 12,000 new cases every year (Saunders et al., 2015). There are no effective therapeutic strategies for SCI that often results in paralysis and sensorimotor impairments. The pathology of SCI can be divided into two stages: the primary injury that is the direct mechanical tissue damage; and the secondary injury that involves a series of biochemical changes, including the disturbances of ionic homeostasis, local hemorrhage, ischemia, edema, free radical stress and inflammatory responses (Penas et al., 2007). Notably, these secondary events could lead to neural apoptosis leading to further persistent damage to the tissue around the injury epicenter (Warden et al., 2001; Bethea and Dietrich, 2002). Previous studies suggest that the stimulation of autophagy plays a significant role in the surviving neurons during secondary injury.

Macroautophagy (hereafter called autophagy) is a catabolic process that regulate the balance of cellular protein synthesis and degradation, in which misfolded and aggregated proteins, lipids and organelles are packaged by double-membrane vesicles, called autophagosomes (APs), and then degraded after fusion with lysosomes (Levine and Klionsky, 2004; Klionsky, 2005; Mizushima, 2007). The entire process by which the autophagic cargo is separated by APs, including the delivery of APs, and subsequent degradation by lysosomal hydrolases after fusion of the APs with lysosomes, is referred to as autophagic flux. Reports suggest that autophagic flux is disrupted following traumatic brain injury (TBI; Sarkar et al., 2014) and SCI (Liu et al., 2015), both of which are associated with neuronal cell death. In addition, impaired autophagic flux frequently results in the accumulation of protein and adult-onset degeneration in cellular implicated in neurodegenerative disorders, such as Alzheimer’s diseases, Huntington’s diseases and Parkinson’s diseases (Nixon et al., 2005; Nixon and Yang, 2011; Nixon, 2013; Sarkar, 2013). Conversely, stimulation of autophagic flux could improve outcomes in animal models of neurodegenerative diseases (Bové et al., 2011) or SCI (Zhang et al., 2017). Thus, the stimulation of autophagic flux has been proposed as a potential therapeutic approach to these conditions.

Transcription factor EB (TFEB) belongs to the MiT family of transcription factors that also includes TFE3, TFEC and MITF (Rehli et al., 1999). Originally associated with renal carcinoma, TFEB is a basic helix-loop-helix leucine zipper transcription factor (Kuiper et al., 2003). Following further in-depth research, TFEB was found to regulate the synthesis of lysosome, by positively regulating genes of the Coordinated Lysosomal Expression and Regulation (CLEAR) network (Sardiello et al., 2009). The CLEAR network consists of genes that are associated with lysosomal hydrolases, lysosomal membrane proteins and components of the vacuolar Hþ-ATPase (v-ATPase) complex (Sardiello et al., 2009; Palmieri et al., 2011). Under normal conditions, TFEB is phosphorylated by mTORC1 on thesurface of lysosome, and this leads to its cytoplasmic sequestration (Settembre et al., 2012). Under other conditions, such as stress and starvation in which mTORC1 is inactivate, mTORC1 is released from the lysosomal surface and can no longer phosphorylate TFEB, resulting in nuclear translocation of TFEB and activation of its target genes (Martina et al., 2012; Roczniak-Ferguson et al., 2012; Settembre et al., 2012). Therefore, the fact that TFEB can be modulated by phosphorylation makes it a promising target for lysosomal biogenesis. Autophagy, which is associated with the processes of degradation and recycling, is strictly relied on lysosomal function (Settembre et al., 2013). Thus, we speculated that regulation of the phosphorylation status of TFEB might influence autophagic flux following SCI.

In mammals, the netrins consist of a series of evolutionarily conserved axon guidance cues that facilitate neurite outgrowth and guide growth cone navigation (Lai Wing Sun et al., 2011). They include four members, namely Netrin-1, Netrin-2, Netrin-3, and β-netrin (Kennedy et al., 1994; Koch et al., 2000). Netrin-1 is initially recognized as a chemotropic factor that attracts or repels axons, lying on which netrin receptor is expressed on the individual axon (Serafini et al., 1996). In recent years, findings have shown that Netrin-1 plays a neuroprotective role following middle cerebral artery occlusion in mice either by decreasing the size of the infarct to enhance recovery (Ding et al., 2014), or attenuating ischemic stroke-induced apoptosis (Wu et al., 2008). In addition, our previous study found that Netrin-1 could stimulate autophagy through inhibition of mTOR via activation of AMP-activated protein kinase (AMPK), a phenomenon that improves the prognosis following SCI in rats (Bai et al., 2017). Based on all of the above findings, we speculated that the stimulation of autophagy by Netrin-1 is due to the promotion of autophagic flux, through the enhancement of lysosomal function via regulation of nuclear localization of TFEB following SCI in rats.

In this study, we aimed to investigate the underlying mechanisms by which Netrin-1 stimulates autophagy and improves functional recovery following SCI. Our results suggested the following: (i) Netrin-1 could enhance lysosomal biogenesis through regulation of the phosphorylation status of TFEB via the AMPK/mTOR signaling pathway in rats after SCI; and (ii) Netrin-1 could inhibit neural apoptosis and improve functional recovery through the promotion of autophagic flux, via the enhancement of lysosomal biogenesis after SCI. Therefore, the modulation of lysosomal biogenesis by the regulation of nuclear localization of TFEB might be a potential strategy for the treatment of SCI.

## Materials and Methods

### Animal Model of Spinal Cord Injury and Groups

We used adult female Sprague-Dawley rats (the Animal Laboratory of Jinzhou Medical University in Jinzhou, China) weighing 220–250 g. The protocol for animal care and use complied with the Guidelines for Animal Experiments, and all procedures were approved by the ethics committee of Jinzhou Medical University. All rats were fed under standard temperature conditions with an alternating 12 h light-dark cycle. Following adaptation to the new environment, 60 rats were randomly divided into four groups: (1) sham group (*n* = 15); (2) SCI group (*n* = 15); (3) Netrin-1 group (*n* = 15); and (4) Netrin-1 + Compound C group (*n* = 15). Anesthesia was achieved following the administration of 10% chloral hydrate (3.0 mL/kg, i.p.). Rats were placed on a cork platform and laminectomies were carried out at the T9 level after the skin was incised. Subsequently, a 10-g impactor device (diameter: 2 mm) was dropped from a height of 2.5 cm onto the surface of exposed spinal cord, and immediately removed without disrupting the intact dura. After rinsing with 0.9% sterile saline, the incisions were sutured, layer by layer. Laminectomy was the only procedure performed on the sham group of rats, none of which sustained any impact injury. Following SCI, the urinary bladder was manually expressed three times daily, until the recovery of bladder function.

### Drug Treatment

Recombinant rat Netrin-1 (Creative Biomart, Shirley, NY, USA) was dissolved in 1× phosphate-buffered saline (PBS) to achieve a final concentration of 800 ng/mL. Compound C (Selleck Chemicals LLC, USA) was dissolved in 1× PBS and administered at a dose of 20 mg/kg (Bai et al., 2017). After SCI, Netrin-1 (1 ml) was immediately administered to the rats in the Netrin-1 group by intraperitoneal injection, and subsequently administered once daily for the next 2 days. After contusion, Netrin-1 (1 mL) and Compound C (20 mg/kg) were immediately administered to the rats of the Netrin-1 + Compound C group by intraperitoneal injection, and again subsequently administered once daily for the next 2 days. Rats in the sham or SCI group were intraperitoneally injected with 1 mL of 1× PBS after surgery, and followed by similar administration once daily for the next 2 days.

### Locomotion Tests

In order to evaluate the recovery of locomotion function after SCI, the Basso, Beattie and Bresnahan (BBB) open field locomotor rating scale (Basso et al., 1995) was introduced. The scale was developed by using the natural progression of locomotion recovery in rats with thoracic SCI (Zhang et al., 2017). In short, the BBB scores range from 0 (complete paralysis) point to 21 points (normal locomotion). The scores were simultaneously evaluated by two trained examiners, who were blinded to the treatment group at 0, 1, 3, 7, 14, 21 and 28 days post-operation. In order to obtain the maximum scores, rats were placed in an open field for 4 min before sacrifice. The mean scores were used for analysis.

### Tissue Preparation

Briefly, animals from each group were again anesthetized with 10% chloral hydrate (3.0 mL/kg, i.p.) at 3 and 28 days post-operation. Rats were then transcardially perfused with 0.9% saline, then perfused with 4% paraformaldehyde in 0.1 M 1× PBS (pH = 7.4). A 2-cm segment of the spinal cord about (rostral and caudal to the epicenter) was removed as soon as possible, and then fixed in 4% paraformaldehyde overnight. The tissue were then immersed in 30% sucrose in 4% paraformaldehyde, until sank to the bottom of the container. Several 10-μm transverse frozen sections were cut using a freezing microtome.

### Western Blot Analysis

At 3 days after SCI, rats were sacrifice with an anesthetic overdose of 10% chloral hydrate. Spinal cord samples (5 mm rostral and caudal to the epicenter) were then rapidly removed and stored at −80°C for Western blotting. The tissues were dissolved in ice-cold radioimmunoprecipitation assay lysis buffer (Beyotime Institute of Biotechnology, China) containing phenylmethylsulfonyl fluoride (PMSF). After spinal cord tissue homogenates were incubated for 20 min on ice and centrifuged at 12,000 rpm, for 30 min at 4°C, the debris was removed. The supernatant was quantified using the Bicinchoninic Protein Assay Kit (Beyotime Institute of Biotechnology, China). The equivalent of 40 μg of total protein was loaded onto the gel for sodium dodecyl sulfate-polyacrylamide gel electrophoresis (SDS-PAGE) and transferred onto a polyvinylidene difluoride membrane (Bio-Rad). Subsequently, the membrane was blocked with 5% non-fat milk in Tris buffered saline with 0.1% Tween-20 for 2 h at room temperature, and then incubated at 4°C overnight with the following primary antibody solutions: anti-light chain 3 (LC3) antibody (1:1000 Abcam, USA); anti-p-AMPK antibody (1:2000 Cell Signaling Technology, Danvers, MA, USA); anti-p-Acetyl-CoA Carboxylase (anti-p-ACC) antibody (1:1000 Cell Signaling Technology, Danvers, MA, USA); anti-p-mTOR antibody (1:2000 Abcam, USA); anti-p-P70S6K antibody (1:1000 Cell Signaling Technology, Danvers, MA„ USA); anti-lysosomal-associated membrane protein 1 (anti-LAMP1) antibody (1:5000; Abcam, USA); anti-Sequestosome 1 (SQSTM1)/p62 antibody (1:400; Abcam, USA); anti-TFEB antibody (1:400; Abcam, USA); anti-Cathepsin D (CTSD) antibody (1:500; Abcam, USA); anti-Bcl-2 antibody (1:1000; Abcam, USA); anti-Bax antibody (1:1000; Abcam, USA); anti-Cleaved-caspase-3 antibody (1:1000 Cell Signaling Technology, Danvers, MA, USA); anti-β-tubulin antibody (1:2000 TransGen Biotech, China); and anti-Histone H3 (1:1000, Beyotime Biotech Inc, China). On the following day, the membranes were incubated at room temperature for 2 h with the corresponding secondary antibodies (1:10,000; EarthOX, Millbrae, CA, USA). Signals of bands were visualized using the ChemiDoc-ItTMTS2 Imager (UVP, LLC, Upland, CA, USA) and densitometric quantification of bands was performed using the ImageJ2x software program (National Institute of Health, New York, NY, USA).

To extract the nuclear proteins, the spinal cord samples were lysed using a nuclear and cytoplasmic protein extraction kit (Beyotime Biotech Inc, China), according to the manufacturer’s instructions. The supernatants were collected as the cytoplasmic fraction, after tissue lysates were centrifuged at 12,000 *g* for 5 min at 4°C. The pellets were then resuspended in a buffer containing 1 mM PMSF for 30 min at 4°C. After the lysates were centrifuged at 12,000 *g* for 10 min, the supernatants containing the nuclear proteins were collected for western blot analysis. Histone H3 was measured as the control for the nuclear compartment.

### Immunofluorescence Analysis

The transverse 10-μm sections, 5 mm rostral to the injury epicenter of each group (*n* = 5) were selected at 3 days after SCI and used for immunofluorescence staining. After the sections were dried at room temperature, they were incubated with a blocking buffer (5% normal goat serum diluted in 0.1% PBS, with Triton X-100) for 2 h at 4°C. The sections were then incubated with the first primary antibody, anti-NeuN antibody (1:500 Abcam, USA; 1:400 Bioss, China) at 4°C overnight. On the next day, the sections were incubated with Alexa Fluor^®^ 488 (1:400; Life Technologies, USA) for 2 h at room temperature. The sections were then incubated with the second primary antibody that included anti-LC3 antibody (1:200 Abcam, USA); anti-LAMP1 antibody (1:20; Abcam, USA); anti-P62 antibody (1:50; Abcam, USA); anti-TFEB antibody (1:100; Abcam, USA); and anti-cleaved-Caspase-3 antibody (1:400 Cell Signaling Technology, Danvers, MA, USA), for 2 h at room temperature, followed by incubation with Alexa Fluor^®^ 568 (1:400; Life Technologies, Carlsbad, CA, USA) for 2 h at room temperature. After re-dyed with 40,6-diamidino-2-phenylindole solution, the sections were sealed with coverslips. All images were captured on a fluorescence microscope (Leica, Germany). The ImageJ2x software was used for the calculation of positive cells and measurement of fluorescence density. The number of double-labeled positive neurons of LC3, P62, cleaved-Caspase-3, or TFEB were counted at 10 randomly chosen fields from each rat and sum up. The total number of positive neurons from each rat were calculated and used for analysis.

### Nissl Staining

The 10-μm transverse frozen sections, resected from 5 mm rostral to the injury epicenter in rats of each group (*n* = 5), were collected for Nissl staining, 28 days post-operation. After being dried, the sections were soaked in a mixture (1:1 alcohol/chloroform) overnight. The next day, after being rehydrated with 100% alcohol, 95% alcohol, and triple-distilled water, the sections were stained with 0.1% Cresyl violet (Sigma-Aldrich, St. Louis, MO, USA) solution. The sections were subsequently differentiated in 95% alcohol, dehydrated in anhydrous alcohol and rinsed in xylene. The number of neurons in the sections of each rat was manually counted under a light microscope. The quantity of ventral motor neurons of the section from each rat were respectively counted under a light microscope at high magnification. Then the mean number of neurons from each rat was calculated and used for subsequent analysis.

### Statistical Analysis

Data were presented as mean ± standard deviation (SD) and analyzed using the SPSS 23.0 software. Comparisons between two groups were performed using the Student’s *t*-test. Comparisons among more than two groups were performed using a one-way analysis of variance (ANOVA) and the Dunnett’s *post hoc* test. *P* values < 0.05 were considered statistically significant.

## Results

### Netrin-1 Promotes Nuclear Translocation of TFEB Via the AMPK/mTOR Signaling Pathway Following SCI

**Figure 1:**
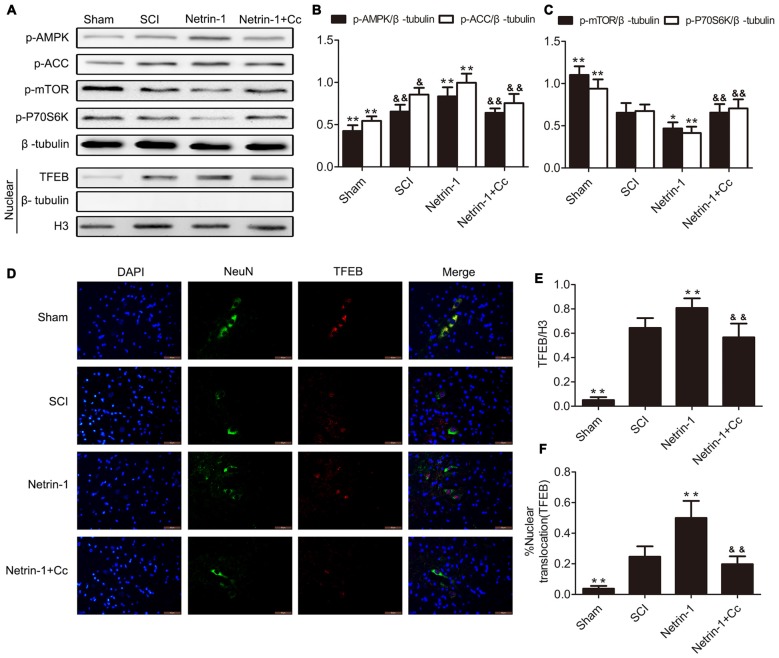
Netrin-1-promotes transcription factor EB (TFEB) nuclear translocation via the AMP-activated protein kinase/mammalian target of rapamycin (AMPK/mTOR) signal pathway after spinal cord injury (SCI). **(A)** Western blots of p-AMPK, p-Acetyl-CoA Carboxylase (p-ACC), p-mTOR, p-P70S6K, TFEB, H3 and β-tubulin in each group. **(B,C,E)** Quantification of p-AMPK, p-ACC, p-mTOR, p-P70S6K, TFEB, H3 and β-tubulin in each group (*n* = 5). **(D)** Double staining for NeuN (green)/TFEB (red) of sections from the spinal cord in each group. **(F)** Quantification of the nuclear-translocation neurons of TFEB in each group (*n* = 5); *P < 0.05 vs. SCI group, **P < 0.01 vs. SCI group, ^&^*P* < 0.05 vs. Netrin-1 group ^&&^*P* < 0.01 vs. Netrin-1 group.

Based on the observation that mTORC1 regulates nuclear translocation of TFEB (Settembre et al., 2012), and our previous findings that Netrin-1 activates the AMPK/mTOR signaling pathway following SCI in rats (Bai et al., 2017), we postulated that Netrin-1 could regulate the nuclear translocation of TFEB via the AMPK/mTOR signaling pathway. ACC and P70S6K are the substrate of AMPK and downstream target of mTOR, respectively, and are representative of the activity of AMPK and mTOR. As our western blot results show (Figures 1A–C), compared to the sham group, a notably higher expression of p-AMPK and p-ACC, accompanied by lower expression of p-mTOR and p-P70S6K was observed in the spinal cord tissue from the SCI group. This indicates that the AMPK/mTOR pathway was activated following SCI. In comparison to the SCI group, treatment with Netrin-1 remarkably enhanced the expression of p-AMPK and p-ACC, but significantly reduced the expression of p-mTOR and p-P70S6K. However, Compound C, an AMPK inhibitor, might have abolished the activation of AMPK by Netrin-1, as reflected by the reduced expression of p-AMPK and p-ACC, as well as the increased expression of p-mTOR and p-P70S6K in the spinal cord tissue of the Netrin-1 + Compound C group in comparison to the Netrin-1 group. This indicated that Netrin-1 could activate the AMPK/mTOR pathway after SCI in rats, and this activation could be abolished by Compound C.

In order to investigate the extent of nuclear translocation of TFEB following SCI, the levels of TFEB in the nuclear compartment were examined using western blot. In the SCI group, significant upregulation of TFEB was observed in the nucleus of neurons with the comparison of Sham group (Figures 1A,E). Compared to the SCI group, treatment with Netrin-1 considerably increased the expression of TFEB in the nucleus of neurons, an increase that was abolished by Compound C. Moreover, immunofluorescence staining of TFEB was also performed, and further examination under immunofluorescence microscopy revealed that neurons in which nuclear translocation (green) of TFEB occurred, were identified with an overlap of red TFEB and a blue nucleus (Figure 1D). Consistent with the results of western blot analysis, the ratio of neurons in which nuclear translocation of TFEB occurred, was remarkably increased in the SCI group, compared to the sham group. However, there is significant difference between the SCI group and the Netrin-1 + Compound C groupin the ratio of neurons in which translocation of TFEB was observed. These findings indicate that nuclear translocation of TFEB occurred after SCI in rats, and Netrin-1 might promote this translocation via the AMPK/mTOR signaling pathway. However, these effects were abolished by Compound C.

### Netrin-1 Enhances Lysosomal Biogenesis Following SCI

**Figure 2:**
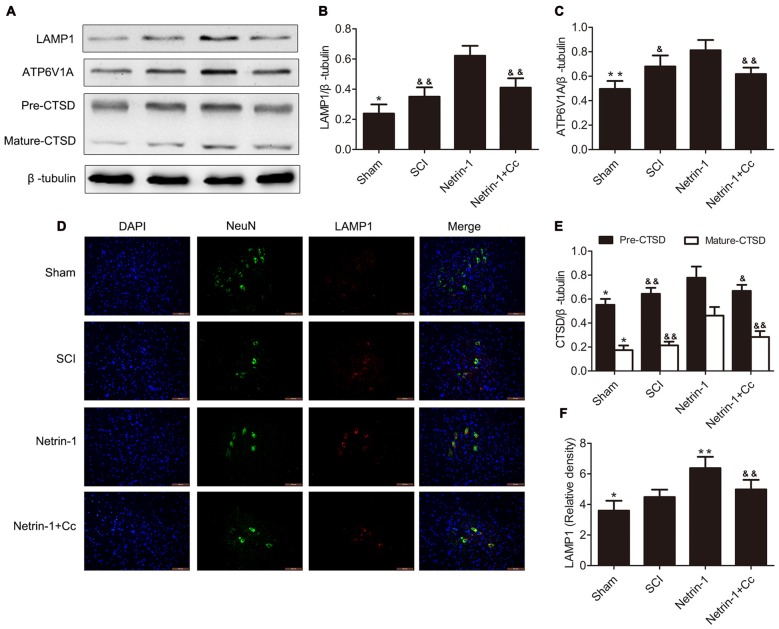
Netrin-1 enhances lysosomal biogenesis after SCI. **(A)** Western blots of lysosomal-associated membrane protein 1 (LAMP1), ATP6V1A, CTSD (Pre-CTSD and Mature-CTSD) and β-tubulin in each group. **(B,C,E)** Quantification of LAMP1, ATP6V1A, CTSD (Pre-CTSD and Mature-CTSD) and β-tubulin in each group (*n* = 5). **(D)** Double staining for NeuN (green)/LAMP1 (red) of sections from the spinal cord in each group. **(F)** Quantification of the number of LAMP1-positive neurons in each group (*n* = 5); *P < 0.05 vs. SCI group, **P < 0.01 vs. SCI group, ^&^*P* < 0.05 vs. Netrin-1 group ^&&^*P* < 0.01 vs. Netrin-1 group.

To investigate the effects of regulating lysosomal biogenesis with Netrin-1 treatment, the expression of LAMP1, CTSD and ATP6V1A was detected in samples of spinal cord tissue, using western blot or immunofluorescence after SCI. In comparison to the sham group, significantly higher expression of LAMP1 was observed in the tissue samples of the SCI group. In comparison to the SCI group, the group treated with Netrin-1 showed remarkably increased expression of LAMP1. However, this change was abolished by Compound C in the Netrin-1 + Compound C group (Figures 2A,B). Consistent with these findings, the immunofluorescence results showed that the fluorescence intensity of LAMP1 was significantly increased after SCI and Netrin-1 further enhanced this intensity. However, this enhancement was abolished by Compound C (Figures 2D,F).

Furthermore, the changing trends in the expression of CTSD (both full length CTSD and mature CTSD) and ATP6V1A among these groups was roughly the same as that of LAMP1. To summarize, these findings indicate that lysosomal biogenesis was upregulated after SCI and further enhanced following treatment with Netrin-1; however, these effects were abolished by Compound C.

### Netrin-1 Promotes Autophagic Flux after SCI

**Figure 3:**
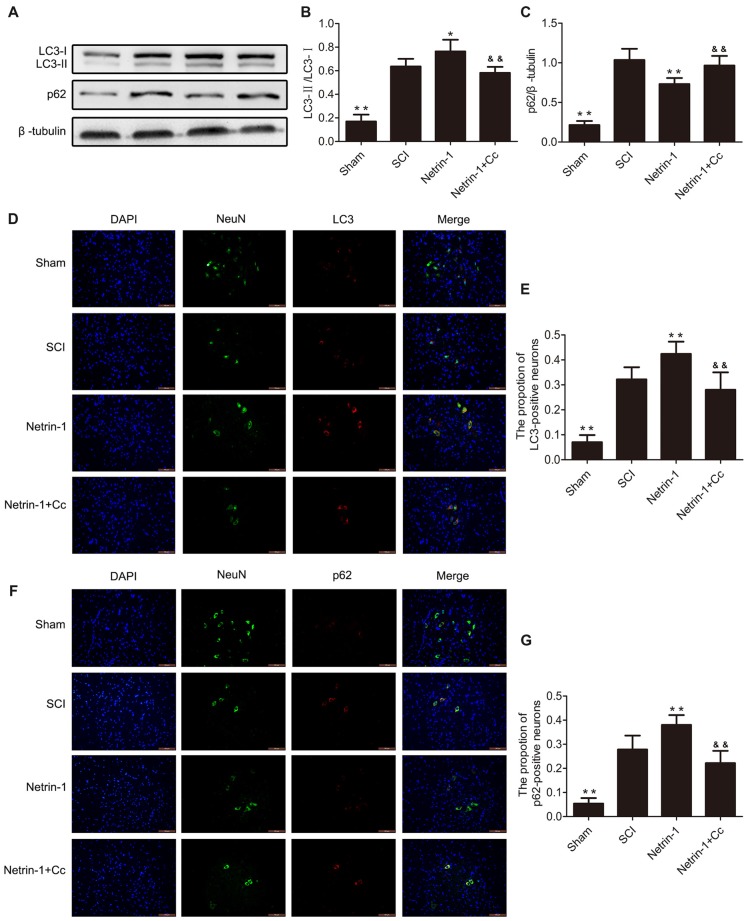
Netrin-1 promotes autophagy flux after SCI. **(A)** Western blots of light chain 3 (LC3)-I, LC3-II, p62 and β-tubulin in each group. **(B,C)** Quantification of LC3-I, LC3-II, p62 and β-tubulin in each group (*n* = 5). **(D)** Double staining for NeuN (green)/LC3 (red) of sections from the spinal cord in each group (white arrows: LC3-positive neurons). **(E)** Quantification of the number of LC3-positive neurons in each group (*n* = 5). **(F)** Double staining for NeuN (green)/p62 (red) of sections from the spinal cord in each group. **(G)** Quantification of the number of p62-positive neurons in each group (*n* = 5). *P < 0.05 vs. SCI group, **P < 0.01 vs. SCI group; ^&&^*P* < 0.01 vs. Netrin-1 group.

To assess autophagic flux in the injured spinal cord, immunofluorescence staining and western blot analysis for LC3 and p62 were both performed (Figures 3A–G). The protein LC3 is known as a marker of autophagy, and the conversion of LC3-I to LC3-II plays a significant role in the formation of APs. The adapter protein p62 is involved in mediating the delivery of ubiquitinated cargo to APs and is degraded by the lysosome together with the autophagic cargo. The levels of p62 represent the degradation rate of APs, and when considered in combination with levels of LC3, can reflect the extent of autophagic flux within cells. When autophagic flux is induced, the number of APs and level of LC3-II both increased; however, the levels of p62 were reduced. When autophagic flux is blocked, the levels of both LC3-II and p62 show an increase (Lipinski et al., 2015). Our Western blot suggested showed that the expression of LC3-II and p62 were remarkably increased following SCI. Furthermore, a remarkably higher ratio of LC3-positive neurons and p62-positive neurons were simultaneously observed after immunofluorescence staining. These findings indicate that autophagic flux was blocked in the injured spinal cord tissue. with the comparision of the SCI group, the group treated with Netrin-1 showed an obvious increase in the levels of LC3-II, but a significant reduction in levels of p62. Moreover, a significantly higher ratio of LC3-positive neurons and a lower ratio of p62-positive neurons were observed in the Netrin-1 group. These findings suggest that treatment with Netrin-1 promotes autophagic flux by simultaneously upregulating both the formation of APs and their degradation rates. Moreover, it is noteworthy that promotion of autophagic flux by Netrin-1 was inhibited by Compound C, as evidenced by significantly higher levels of p62 and considerably lower levels of LC3 in the results of both western blot and immunofluorescence analyses. Based on these results, treatment with Netrin-1 promotes autophagic flux following SCI in rats; however, this effect is abolished by Compound C.

### Netrin-1 Inhibits Neuronal Apoptosis after SCI

**Figure 4:**
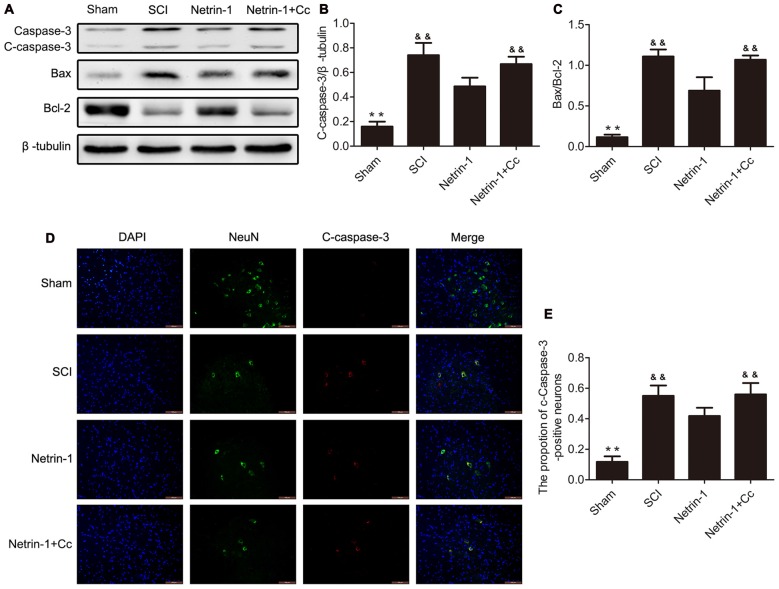
Netrin-1 attenuates neural apoptosis after SCI. **(A)** Western blots of C-caspase 3, Bax, Bcl-2 and β-tubulin in each group. **(B,C)** Quantification of C-caspase 3, Bax, Bcl-2 and β-tubulin in each group (*n* = 5). **(D)** Double staining for NeuN (green)/ C-caspase 3 (red) of sections from the spinal cord in each group. **(E)** Quantification of the number of C-caspase 3-positive neurons in each group (*n* = 5); **P < 0.01 vs. SCI group; ^&&^*P* < 0.01 vs. Netrin-1 group.

To test whether treatment with Netrin-1 attenuates neuronal apoptosis, the expression of the apoptotic protein cleaved-caspase 3, antiapoptotic protein Bcl-2, and proapoptotic protein Bax were detected by western blot and immunofluorescence staining. Western blot results showed that treatment with Netrin-1 remarkably reversed the increased levels of cleaved-caspase 3 and the ratio of Bax/Bcl-2 after SCI; however, Compound C restored these reductions induced by Netrin-1 in the injured spinal cord, based on the results of the Netrin-1 + Compound C group (Figures 4A–C). In addition, the ratio of cleaved-caspase 3-positive neurons was remarkably increased after SCI and Netrin-1 significantly reduced the ratio of cleaved-caspase 3-positive neurons; however, this reduction was restored by Compound C (Figures 4D,E). These findings suggest that administration of Netrin-1 effectively inhibited neuronal apoptosis after SCI in rats; however, this antiapoptotic effect was abolished by Compound C.

### Netrin-1 Reduces the Loss of Motor Neurons Following SCI

**Figure 5:**
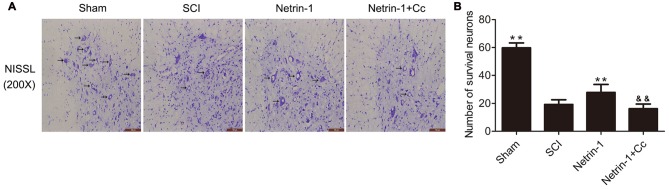
Netrin-1 decreases the loss of neurons after SCI. **(A)** Nissl staining of sections from the spinal cord in each group at 28 days after SCI, Black arrows: surviving neurons. **(B)** Quantification of surviving neurons in each group (*n* = 5); **P < 0.01 vs. SCI group; ^&&^*P* < 0.01 vs. Netrin-1 group.

To determine whether Netrin-1 reduces the loss of motor neurons following SCI, Nissl staining was performed to facilitate the counting of surviving motor neurons in the anterior horn of the spinal cord (Figures 5A,B). with the comparision of sham group, the number of motor neurons in the anterior horns of the injured spinal cords was significantly reduced in the SCI group. Compared to the SCI group, the group treated with Netrin-1 showed remarkable survival of a greater number of motor neurons. Notably, in comparison to the Netrin-1 group, the combined application of Netrin-1 and Compound C significantly reduced the numbers of surviving motor neurons. These findings indicate that Netrin-1 reduces the loss of motor neurons after SCI, but this neuroprotective effect is abolished by Compound C.

### Netrin-1 Improves Functional Recovery after SCI

**Figure 6:**
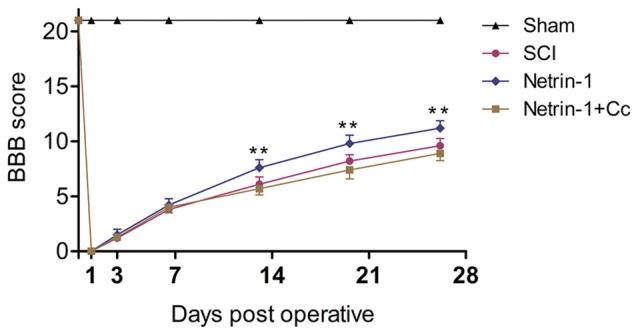
Netrin-1 improves functional recovery after SCI. Basso, Beattie and Bresnahan (BBB) scores of rats in each group were evaluated at 0, 1, 3, 7, 14, 21 and 28 days after operation. **P < 0.01 vs. SCI group.

To evaluate the effects of Netrin-1 treatment on locomotor recovery after SCI, the BBB scores were measured at different time points during the 4 weeks following the operation (Figure 6). Our results showed that the rats in the sham group had normal BBB scores and the scores of rats in the other groups were all significantly below normal for the duration of the investigation. There is no statistical difference between the BBB scores of rats in the SCI group and those in the Netrin-1 + Compound C group at all time points. In addition, the BBB scores of rats in the Netrin-1 group were significantly higher than those in the SCI group from 14 days after contusion. These results suggest that Netrin-1 improves functional recovery after SCI in rats, but this effect is abolished by Compound C.

## Discussion

In this study, our results demonstrated that treatment with Netrin-1 enhances lysosomal biogenesis by promoting nuclear translocation of TFEB via the AMPK/mTOR signaling pathway in rats after SCI. Our findings also suggest that the enhancement of lysosomal biogenesis by Netrin-1 could promote autophagic flux and improve the prognosis following SCI. This could partly explain the neuroprotective effect of Netrin-1 treatment after SCI, reported in our previous study (Bai et al., 2017). To our best knowledge, this is the first study to investigate the underlying mechanisms by which Netrin-1 stimulates autophagy and improves the prognosis following SCI.

A SCI is a devastating condition that often leads to severe and permanent neurological deficits. It is caused by direct mechanical impact on spinal cord tissue and this damage is referred to as the primary injury. This injury can lead to a series of biochemical events that can cause either more delayed or progressive cell loss that is referred to as the secondary injury (Beattie et al., 2002). Although the details of the mechanisms underlying the biochemical events in secondary injury remain unclear, blocking or reducing the levels of secondary neuronal death at that time might reduce the associated disabilities (Cavallucci and D’Amelio, 2011; Li et al., 2016). Therefore, it would be beneficial to have more focused research on the biochemical events that might inhibit neuronal apoptosis during secondary injury.

Netrin-1, a member of the netrin family, is expressed in the central nervous system during embryonic development, and in neurons, as well as oligodendrocytes in the spinal cord of adult rats (Manitt et al., 2001). Netrin-1 is initially recognized as a chemotropic factor that either attracts or repels axon outgrowth via interaction with different netrin receptors that are expressed in the individual axon (Serafini et al., 1996). With increasing developments in research, the neuroprotective effect of Netrin-1 was discovered, evidenced by the reduction in infarct size or attenuation of ischemic stroke-induced neuronal apoptosis to promote angiogenesis in mice after middle cerebral artery occlusion (Wu et al., 2008; Ding et al., 2014). Previous studies report that Netrin-1 could activate AMPK to induce neurite outgrowth in cortical neurons by increasing the phosphorylation of AMPK via interaction with its receptor, Down syndrome cell adhesion molecule (DSCAM). Furthermore, the activated AMPK might affect the actin cytoskeleton by inhibiting mTOR (Dasgupta and Milbrandt, 2007; Zhu et al., 2013). This shows Netrin-1 could activate the AMPK/mTOR signaling pathway by interacting with its receptor DSCAM in cortical neurons. In the present study, our western blot results showed that treatment with Netrin-1 remarkably increased the expression of p-AMPK and p-ACC, and simultaneously significantly reduced the levels of p-mTOR and p-P70S6K following SCI. These findings suggest that Netrin-1 could activate the AMPK/mTOR signaling pathway after SCI. However, the administration of Compound C, a selective AMPK inhibitor, abolished the activation induced by Netrin-1, as reflected by the significantly increased levels of p-mTOR and p-P70S6K, accompanied by the reduced levels of p-AMPK and p-ACC. These results indicate that Netrin-1 indeed inactivated mTOR by activating AMPK. These findings are consistent with those of our previous report (Bai et al., 2017).

As a novel transcription factor, TFEB has been identified as a member of the MiT family. Previous studies report that TFEB could control lysosomal biogenesis by positively regulating genes of the CLEAR network that consists of genes associated with lysosomal hydrolases, lysosomal membrane proteins and components of the vacuolar Hþ-ATPase (v-ATPase) complex (Sardiello et al., 2009; Palmieri et al., 2011). Under normal conditions, mTORC1 promotes the binding of TFEB to 14-3-3 proteins, thereby preventing the nuclear translocation and activation, following the phosphorylation of TFEB on the lysosomal surface (Martina et al., 2012; Roczniak-Ferguson et al., 2012; Settembre et al., 2012). During starvation or other stress conditions, mTORC1 dissociates from the lysosomal surface, leading to TFEB dephosphorylation and nuclear translocation that effectively upregulates the transcription of its target genes (Settembre et al., 2011; Shin et al., 2016; Medina et al., 2017). For example, research studies have shown that the expression of TFEB target genes is upregulated after mTORC1 activity is inhibited by treatment with chloroquine in the hepatocytes of mice (Settembre et al., 2012). We know that mTOR is the catalytic subunit of the complex-mTOR complex 1(mTORC1). The enzyme AMPK responds to energy stress in part through its inhibition of the mTOR pathway (Gwinn et al., 2008).

As our results show in Figures 1, 2, compared with the SCI group, the group treated with Netrin-1 had significantly enhanced ratios of neurons in which nuclear translocation of TFEB was observed, and simultaneously exhibited increased expression of LAMP1, ATP6V1 and CTSD following SCI (Figures 1, 2). However, the combined administration of Netrin-1 and Compound C remarkably inhibited the nuclear translocation of TFEB and expression of LAMP1, ATP6V1 and CTSD in comparison to the Netrin-1 group. In addition, treatment with Netrin-1 significantly activated the AMPK/mTOR signaling pathway after SCI, and this activation was reversed by Compound C (Figures 1A–C). These findings suggest that Netrin-1 might enhance lysosomal biogenesis through promotion of nuclear translocation of TFEB via activation of the AMPK/mTOR pathway following SCI.

Autophagy is a lysosome-dependent catabolic process that degrades aggregated protein, damaged organelles and cytoplasmic proteins (Sarkar et al., 2005; Caccamo et al., 2010; Yang and Klionsky, 2010). Autophagic flux refers to the process by which the cargo is separated by the Aps, and includes the delivery of APs, and subsequent degradation by lysosomal hydrolases, after APs are fused with the lysosomes. Thus, it is not difficult to see that autophagic flux is strictly dependent on lysosomal function. For example, previous studies report that lysosomal dysfunction contributes to the disruption of autophagic flux in rats with SCI (Liu et al., 2015) or TBI (Sarkar et al., 2014). The LC3-II protein can be detected as a marker of the autophagosome; furthermore, p62 directs ubiquitinated cargo to APs for degradation, and is degraded together with its cargo (Lipinski et al., 2015; Zhang et al., 2017). The levels of p62 can reflect the degree of degradation of APs and cell clearance. It remains unclear, at least in TBI, whether the accumulation of APs after trauma is due to an increase in APs biosynthesis and elevation of autophagic flux, or to a reduction in AP degradation and inhibition of flux (Lipinski et al., 2015). As our results show in Figure 3, compared to the SCI group, the group treated with Netrin-1 showed a significant increase in the expression of LC3-II and the ratio of LC3-positive neurons, as well as a remarkable reduction in the levels of p62 and ratio of p62-positive neurons. Generally, Netrin-1 both upregulated AP biosynthesis and increased the degradation rate of APs in neurons, indicating that Netrin-1 elevated autophagic flux in the injured spinal cord. However, in comparison to the SCI group, the combined administration of Netrin-1 and Compound C abolished the elevated autophagic flux induced by Netrin-1, as reflected by the lack of statistical significance in the expression of p62 and LC3-II. At the same time, it is noteworthy that Netrin-1 significantly enhanced lysosomal biogenesis after SCI; however, this effect was also abolished by treatment with Compound C. These results suggest that Netrin-1 could promote autophagic flux by enhancing lysosomal biogenesis, via activation of the AMPK/mTOR pathway after SCI. Moreover, the effect of upregulating the formation of APs by Netrin-1 cannot be ignored when the elevation of autophagic flux is considered. Interestingly, one study found that TFEB can be phosphorylated by mTOR at serine 211 (S211), and the mutation of S211 to alanine (TFEB-S211A) causes significant accumulation of TFEB in the nucleus of most ARPE-19 cells and induces AP formation, despite the fact that mTORC1 remains active (Martina et al., 2012). In addition, TFEB could coordinate autophagy by positively regulating the formation of APs (Settembre et al., 2011). Therefore, we speculated that Netrin-1 could elevate autophagic flux by inducing AP formation, via the promotion of nuclear translocation of TFEB following SCI in rats. However, the details of the underlying mechanisms remain unclear and require further study.

Autophagic flux plays a major role in cell homeostasis, and seems particularly important in terminally differentiated cells, such as neurons (Hara et al., 2006; Komatsu et al., 2006). Upregulated autophagic flux often improves locomotor function recovery and reduces the apoptosis of neurons in traumatic injury of the central nervous system. For example, metformin could improve functional recovery and attenuate apoptosis caused by SCI by promoting autophagic flux (Zhang et al., 2017). In addition, rapamycin promotes functional recovery and inhibits apoptosis by stimulating autophagic flux in diabetic rats following SCI (Zhou et al., 2015). In contrast, disrupted autophagic flux is correlated with the induction of apoptosis following SCI in rats (Liu et al., 2015), and impaired autophagy contributes to neuronal death after TBI in rats (Sarkar et al., 2014). The Bcl-2 protein exerts an antiapoptotic effect and Bax is involved in the induction of apoptosis (Yang et al., 1997; Wei et al., 2001). Cleaved-caspase-3 is a hallmark of apoptotic cell death that performed as the final executor of apoptosis (Min et al., 2014). Our western blot analyses revealed remarkably higher levels of C-caspase 3 and a significantly higher ratio of Bax/Bcl-2 in the SCI group, in comparison to the Netrin-1 group (Figure 4). Furthermore, double staining (for NeuN/C-caspase 3) showed that Netrin-1 significantlydecreased the percentage of C-caspase 3-positive neurons in the injured spinal cord, in comparison to the SCI group. These findings indicate that Netrin-1 attenuated neuronal apoptosis in the injured spinal cord. However, no significant differences were noted between the SCI group and the Netrin-1 + Compound C group, in the expression of C-caspase 3 and ratio of Bax/Bcl-2, as well as the percentage of C-caspase 3-positive neurons in the injured spinal cord. This suggests that Compound C abolished the antiapoptotic effect of Netrin-1 treatment following SCI. These findings also suggest that Netrin-1 might attenuate neuronal apoptosis in the injured spinal cord by promoting autophagic flux. Consistent with the anti-apoptotic effect of Netrin-1, the results of Nissl staining and BBB scores showed that Netrin-1 significantly increased the number of surviving neurons in the anterior horn of the spinal cord and remarkably enhanced the BBB score after SCI. However, no significant differences were noted in the number of surviving neurons and the BBB scores between the SCI group and the Netrin-1 + Compound C group. Taken together, these data suggest that Netrin-1 possibly inhibits neuronal apoptosis, reduces the loss of motor neurons, and improves functional recovery by promoting autophagic flux after SCI.

We also noted that TFEB could regulate autophagy through AP-lysosome fusion (Settembre et al., 2011). Therefore, to some extent, the nuclear translocation of TFEB in neurons might also contribute to the promotion of autophagic flux through AP-lysosome fusion after SCI in rats. Therefore, one of the limitations of the present study is the fact that the underlying mechanisms, by which Netrin-1 treatment regulated AP formation and AP-lysosome fusion, were not elaborated. Furthermore, lacking evidence from *in vitro* experiments is another limitation. These limitations therefore guide the direction of the next steps of our research.

In conclusion, our results suggest that: (i) Netrin-1 could enhance lysosomal biogenesis by regulating the phosphorylation status of TFEB via the AMPK/mTOR signaling pathway following SCI in rats; (ii) Netrin-1 could elevate autophagic flux by promoting the degradation of APs via the enhancement of lysosomal biogenesis, and upregulating the formation of APs; however the associated mechanisms remain unclear; and (iii) Netrin-1 could inhibit neuronal apoptosis and improve functional recovery by promoting autophagic flux following SCI. Therefore, the modulation of lysosomal biogenesis by regulating the nuclear localization of TFEB might be a potential strategy for the treatment of SCI.

## Author Contributions

GLv and LB designed and supervised the research. LB, YB, YY, YW, HW, GLi and PY performed this research. XM analyzed data. LB wrote the manuscript text. All authors reviewed the manuscript.

## Conflict of Interest Statement

The authors declare that the research was conducted in the absence of any commercial or financial relationships that could be construed as a potential conflict of interest.
